# Drug-drug-interactions in patients with atrial fibrillation admitted to the emergency department

**DOI:** 10.3389/fphar.2024.1432713

**Published:** 2024-10-15

**Authors:** Thorsten Bischof, Fiona Nagele, Marius M. Kalkofen, Maximilian E. O. Blechschmidt, Hans Domanovits, Markus Zeitlinger, Christian Schoergenhofer, Filippo Cacioppo

**Affiliations:** ^1^ Department of Clinical Pharmacology, Medical University of Vienna, Vienna, Austria; ^2^ Department for Medicines Information and Clinical Pharmacy, Pharmacy of The University Hospital Vienna, Vienna, Austria; ^3^ Department of Emergency Medicine, Medical University of Vienna, Vienna, Austria

**Keywords:** atrial fibrillation, emergency department, drug-drug-interactions, qtc-prolongation, bleeding

## Abstract

**Introduction:**

Polypharmacy is a growing concern in healthcare systems. While available data on potential drug-drug interactions (pDDI) from emergency department (ED) patients is derived from heterogenous populations, this study specifically focused on patients with atrial fibrillation (AF). We hypothesized that patients with AF have similar comorbidities, receive similar drugs, and have similar pDDIs. The overarching aim was to highlight frequent pDDIs, providing practical guidance for treating healthcare professionals and consequently reduce the risk of adverse drug reactions.

**Methods:**

Two hundred patients ≥18 years with AF, who received rate- or rhythm-controlling medication at the ED of the University Hospital Vienna, and who were on long-term medication before admission, were eligible. Long-term medication alone, as well as in combination with medication administered at the ED were analyzed for pDDIs using the Lexicomp^®^ Drug interactions database.

**Results:**

Within the long-term medication of patients’, we identified 664 pDDIs. Drugs administered at the ED increased pDDIs more than 3-fold to 2085. Approximately, every fifth patient received a contraindicated drug combination (on average 0.24 per patient), while 70% received drug combinations for which therapy modifications are recommended (on average 1.59 per patient). The most frequently involved drugs included amiodarone, propofol, bisoprolol, enoxaparin, and acetylsalicylic acid. Increased risk of bleeding, QTc prolongation, and myopathy were among the most relevant potential consequences of these interactions.

**Discussion:**

In conclusion, an optimization of medication would be advisable in almost every AF patient. Treating healthcare professionals should be cautious of drugs that increase bleeding risk, prolong QTc, or bear a risk for myopathy.

## 1 Introduction

Polypharmacy is a growing problem in healthcare systems of developed countries, which is associated with an increased risk of drug-drug-interactions (DDI) potentially causing adverse drug reactions (ADR) (Muhic et al., 2017; Zheng et al., 2018; Cascorbi, 2012).

The emergency department (ED) is a particularly challenging work environment: healthcare professionals face a heterogenous cohort of patients with complex medical conditions that may be time-critical and require quick decisions (Gonzalez Morganti et al., 2013). Often, patients are neither able to provide a detailed medical history nor information on their current medication (Cooksley et al., 2018). In this setting a thorough and often time-consuming analysis of potential DDIs (pDDI) including the use of clinical decision support systems (CDSS) may be difficult. Thus, treatment is often administered without consideration of pDDIs, although the clinical consequences of such interactions may be significant (Gonzalez Morganti et al., 2013; Vogel et al., 2022).

Atrial fibrillation (AF) is the most common cardiac arrhythmia in adults, with an estimated prevalence of 2%–4% in Europe (Hindricks et al., 2021). Several substances regularly used for the treatment of AF are known to have a relevant interaction potential, including anticoagulants and antiarrhythmic medication. Moreover, patients with AF are also likely to have several comorbidities and therefore, polypharmacy may be a common finding in this cohort (Hindricks et al., 2021).

Previous studies analyzing pDDIs in EDs included large cohorts of elderly patients independent of their diagnosis (Marino et al., 2016; Letinier et al., 2022). These studies provided important data on pDDIs in this specific setting but their impact on prescribers and on clinical routine may have been limited by their study design. In this study, we sought to find an approach that provides practical guidance for treating healthcare professionals. Patients with a specific diagnosis like AF may often have similar underlying comorbidities and, as a consequence, comparable long-term medication. Thus, specific pDDIs may occur repeatedly.

The aim of this study was to identify specific pDDIs that occur frequently in patients being treated for AF in the ED. Identifying these frequent pDDIs may raise awareness, provide practical guidance, and consequently reduce the number of pDDIs in patients with AF taking the special circumstances of the ED into account.

## 2 Methods

This is a retrospective cohort study that included patients, who were treated for AF at the Department of Emergency Medicine of the University Hospital Vienna. Prior to this study, there were no data on pDDIs in this cohort. To obtain a realistic estimate on the frequency and the severity of pDDIs, we included 200 patients with AF retrospectively starting from October 2019.

Patients ≥18 years of age with AF, who received rate or rhythm controlling medication at the ED and who were on long-term medication prior to their ED visit, were eligible for this study. Lexicomp^®^ Drug interactions database, available at www.uptodate.com, was used to identify pDDIs. The database only evaluates active ingredients and provides information about pDDIs divided in five categories: avoid combination (X), consider therapy modification (D), monitor therapy (C), no action needed (B) and no known interaction (A).

Data on long-term medication and medicines prescribed at the ED (e.g., total number of drugs, drug classes, etc.), as well as demographics (age, gender) and other relevant medical data (e.g., admission diagnosis, comorbidities, etc.) were extracted from electronic patient charts and analyzed by non-parametric descriptive statistics (e.g., median, interquartile range (IQR)). The total number of pDDIs, as well as the severity grading according to the above-mentioned categories were retrieved from the Lexicomp database. In addition, we calculated the mean number of pDDIs per patient. We investigated correlations between the total number of drugs, age, gender, and the total number of pDDIs calculating the bivariate Spearman’s rank correlation. Furthermore, the most frequently prescribed drugs were categorized into Anatomical Therapeutic Chemical (ATC) codes and the most common involved drug pairs for pDDIs were presented by descriptive statistics. Moreover, we differentiated between pDDIs concerning the long-term medication of patients and pDDIs that occurred due to additional drug therapy administered in the ED.

A follow-up analysis investigated whether the patients (i) were discharged home after the ED visit, (ii) stayed overnight in the ED and were discharged home the next day, (iii) were admitted to another ward, or (iv) admitted to an intensive care unit. Furthermore, the electronic patient charts were reviewed for clinical events that may have been associated with the identified pDDIs. The follow-up period was approximately 5 years.

In addition, a multidisciplinary team of 6 independent experts (2 ED physicians, 1 clinical pharmacologist, and 3 clinical pharmacists) conducted a comprehensive external review with two major objectives. First, the experts evaluated the most frequently identified X- and D-graded pDDIs (Table 4) of this study. To obtain an overall clinical impression of each patient, the expert team received the diagnosis AF, kidney function (glomerular filtration rate (GFR) and creatinine level), comorbidities, long-term-medication, medication received in the ED, and the possible consequence of each pDDI. The clinical significance of those pDDIs were rated from each expert with the following scale: 0 = no known interaction, 1 = no action needed, 2 = monitor therapy, 3 = therapy modification, 4 = avoid combination. The results of the expert review are presented by descriptive statistics (Table 4). Second, the experts evaluated the medication list of a randomly selected subset of 20 (10%) patients. Each expert reviewed the patients’ medication (long-term medication and the medication received in the ED) for pDDIs using resources of their choice. Based on their assessment, the experts rated the pDDIs with following scoring system: A = unknown interaction, B = no intervention required, C = monitor therapy, D = modify therapy, X = avoid combination. Afterwards, the expert review results were compared descriptively with the Lexicomp^®^ Drug interaction database results (Supplementary Table S1). The study was approved by the local ethics committee (Ethics committee of the Medical University of Vienna, EK-number 2084/2019).

Drugs were categorized into ATC codes: alimentary tract and metabolism (A), blood and blood forming organs (B), cardiovascular system (C), dermatologicals (D), genito urinary system and sex organs (G), systemic hormonal preparations, exclusive sex hormones and insulins (H), anti-infectives for systemic use (J), antineoplastic and immunomodulating agents (L), musculo-skeletal system (M), nervous system (N), antiparasitic products, insecticides and repellents (P), respiratory system (R), sensory organs (S), various (V).

## 3 Results

Two-hundred eligible patients, of whom 105 were women, with a median age of 71 years (IQR 61–77), treated between January 2018 and October 2019 were included in this study. The most frequent comorbidities included hypertension (118 patients) and hyperlipidemia (41 patients). Of 1,576 prescribed substances (median 7; IQR 5–10 per patient), 1,018 substances (64.6%; median 5; IQR 3–7 per patient) belonged to long-term medication, whereas 558 (35.4%; median 3; IQR 2–4 per patient) were administered during the ED visit. Within the patients’ long-term medication, we identified 664 pDDIs (median 2; IQR 0–4 per patient) across all severities (A, B, C, D, X). The number of total pDDIs increased by 1,421 (median 6; IQR 2–10 per patient) to a total of 2085 pDDIs (median 7; IQR 3–13 per patient), when the medication the patients received during their ED stay was added to the analysis. While all patients were admitted to the ED for at least some hours, 102 patients were discharged after having received treatment for AF, 59 patients were hospitalized at the ED overnight and discharged on the next day, 38 patients were admitted to normal wards for further treatment and one patient was admitted to the intensive care unit. Table 1 presents demographics, comorbidities, total number of substances and pDDIs, and drug classes involved in pDDIs. Table 2 presents the distribution of pDDIs over the severity graded C, D and X, as well as the number of patients with at least one pDDI.

**TABLE 1:** Demographics, comorbidities, medication, and pDDIs including all severities of the cohort.

Demographic data	Results
Patients [n= (%)]	200 (100)
Female [n= (%)] Male [n= (%)]	105 (52.5) 95 (47.5)
Age, median (IQR)	71 (61–77)
Creatinine mg/dL, median (IQR)	0.97 (0.81–1.17)
Glomerular Filtration Rate mL/min, median (IQR)	70 (55–85)
Most frequent comorbidities [n= (%)]	
- Hypertension [n= (%)]	118 (59)
- Hyperlipidemia [n= (%)]	41 (21)
- Diabetes mellitus II [n= (%)]	38 (19)
- Coronary artery disease [n= (%)]	31 (16)
- Chronic kidney disease [n= (%)]	19 (10)
total number of drugs (median per patient, IQR)	1,576 (7, 5–10)
- long-term medication (median per patient, IQR)	1,018 (5, 3–7)
- ED medication (median per patient, IQR)	558 (3, 2–4)
drug classes of prescribed drugs [n= (%)]	1,576 (100)
- Alimentary tract and metabolism (A) [n= (%)]	142 (9)
- Blood and blood forming organs (B) [n= (%)]	262 (17)
- Cardiovascular system (C) [n= (%)]	768 (49)
- genito urinary system and sex organs (G) [n= (%)]	14 (1)
- systemic hormonal preparations, exclusive sex hormones and insulins (H) [n= (%)]	48 (3)
- anti-infectives for systemic use (J) [n= (%)]	22 (1)
- antineoplastic and immunomodulating agents (L) [n= (%)]	11 (1)
- musculo-skeletal system (M) [n= (%)]	23 (1)
- nervous system (N) [n= (%)]	217 (14)
- antiparasitic products, insecticides and repellents (P) [n= (%)]	2 (0)
- respiratory system (R) [n= (%)]	63 (4)
- sensory organs (S) [n= (%)]	3 (0)
- various (V) [n= (%)]	1 (0)
total number of pDDIs [n= (median per patient, IQR)]	2085 (7, 3–13)
- pDDIs for long-term medication [n= (median per patient, IQR)]	664 (2, 0–4)
- pDDIs for ED plus long-term medication [n= (median per patient, IQR)]	1,421 (6, 2–10)

ED, emergency department; IQR, interquartile range; pDDIs, potential drug-drug-interactions.

**TABLE 2:** Distribution of pDDIs across severity grades.

Severity	Number of pDDIs (% of total pDDIs)	Number of affected patients with at least one pDDI (% of total pDDIs)	Mean pDDIs per patient
Overall X	48 (2)	37 (19)	0.24
X for long-term medication	17	15 (8)	
X for ED plus long-term medication	31	26 (13)	
Overall D	318 (15)	139 (70)	1.59
D for long-term medication	64	43 (22)	
D for ED plus long-term medication	254	133 (67)	
Overall C	1,555 (75)	180 (90)	7.78
C for long-term medication	494	120 (60)	
C for ED plus long-term medication	1,061	171 (86)	

n = 200, X = avoid combination, D = consider therapy modification, C = monitor therapy; pDDI, potential drug-drug-interaction; ED, emergency department.

Furthermore, the number of pDDIs correlated well with the number of drugs per patient (Spearman’s rank correlation coefficient test r: 0.87; CI 0.83–0.90; p < 0.0001; Supplementary Figure S1). The correlation between pDDIs and age was weak (Spearman’s rank correlation coefficient test r: 0.33; CI 0.20–0.45; p< 0.0001). No correlation was found between pDDIs and gender.

### 3.1 Drugs most frequently involved in pDDIs

Table 3 lists the most common observed drugs involved in pDDIs.

**TABLE 3:** Drugs most frequently involved in pDDIs of severity X, D, C.

Drug	Overall (X-, D-, C- pDDIs)	X-pDDIs	D-pDDIs	C-pDDIs
Amiodarone	552	9	104	439
Propofol	399	1	76	322
Bisoprolol	245	-	7	238
Enoxaparin	150	9	76	65
Acetylsalicylic acid	139	1	61	77
Furosemide	117	-	6	111
Metoprolol	104	-	4	100
Hydrochlorothiazide	103	-	-	103
Amlodipine	85	-	6	79
Metamizole	80	3	31	46
Candesartan	76	-	1	75
Spironolactone	64	1	-	63
Valsartan	63	-	-	63
Ramipril	58	-	1	57
Edoxaban	42	5	11	26
Simvastatin	36	-	26	10

pDDI, potential drug-drug-interaction, X = avoid combination, D = consider therapy modification, C = monitor therapy.

### 3.2 X- and D-graded pDDIs

Special attention was paid to pDDIs with the highest severity grading X and D. The most frequently identified drug pairs of these pDDIs are listed in Table 4. In this context, the most frequently involved drug classes were cardiovascular and neurological drugs. In particular, the combination of edoxaban - enoxaparin was the most prevalent combination among X-graded pDDIs. Amiodarone exhibited a risk of QTc prolongation with various interaction partners over both severity levels. Among D-graded pDDIs, the combinations enoxaparin – acetylsalicylic acid, amiodarone – propofol, and amiodarone - simvastatin were most frequently detected.

**TABLE 4:** Most frequently identified pDDIs of severity rating X and D.

Most frequently identified X-graded pDDIs				
Drug 1	Drug 2	Potential consequence	Frequency	Expert review
Edoxaban	Enoxaparin	increased bleeding risk	5	2
Ipratropium	Tiotropium	anticholinergic effects	4	1
Amiodarone	Quetiapine	risk of QTc prolongation	3	2
Amiodarone	Citalopram	risk of QTc prolongation	3	2
Sotalol	Vernakalant	risk of QTc prolongation	3	2
Ibutilide	Propafenone	risk of QTc prolongation	2	2
Enoxaparin	Rivaroxaban	increased bleeding risk	2	2
Amiodarone	Moxifloxacin	risk of QTc prolongation	2	2
Dronedarone	Vernakalant	risk of QTc prolongation	1	3

Most frequently identified D-graded pDDIs				
Drug 1	Drug 2	Potential consequence	Frequency	Expert review
Amiodarone	Propofol	risk of QTc prolongation	53	1
Acetylsalicylic acid	Enoxaparin	increased bleeding risk	44	2
Amiodarone	Simvastatin	risk of myopathy	17	3
Enoxaparin	Metamizol	increased bleeding risk	10	1
Propofol	Vernakalant	risk of QTc prolongation	9	0.5
Amiodarone	Phenprocoumon	increased bleeding risk	8	2
Acetylsalicylic acid	Metamizol	increased bleeding risk	7	1
Clopidogrel	Enoxaparin	increased bleeding risk	6	2
Amiodarone	Ibutilide	risk of QTc prolongation	6	3
Amlodipin	Simvastatin	risk of myopathy	6	2
Ibutilide	Propofol	risk of QTc prolongation	6	1.5
Tramadol	Propofol	CNS depression	5	1.5

For the expert review a numerical severity grading was applied: 4 = avoid combination, 3 = therapy modification, 2 = monitor therapy 1 = no action needed, 0 = no known interaction. The column expert review presents a median of the individual responses from the six experts. A median of 2, for instance, means that the experts found overall that a specific pDDI, should be monitored; CNS, central nervous system; pDDI, potential drug-drug-interaction.

The potential consequences of pDDIs are shown in Table 5 focusing on X- and D-graded pDDIs. Notably, approximately two-thirds of all X- (avoid combination) and D-graded (consider therapy modification) pDDIs concerned increased risk of bleeding or QTc prolongation. The third most common finding was increased risk of myopathy.

**TABLE 5:** Most frequent potential consequences of X- and D-graded pDDIs.

Potential consequences of X-pDDIs	Results (%)
risk of QTc prolongation	17 (35)
increased bleeding risk	11 (23)
anticholinergic effects	5 (10)
CNS depression	5 (10)
risk of myopathy	2 (4)

Potential consequences of D-pDDIs	Results (%)
Increased bleeding risk	119 (37)
Risk of QTc prolongation	93 (29)
CNS depression	30 (9)
Risk of myopathy	28 (9)

X = avoid combination, D = modify therapy, CNS, central nervous system; pDDI, potential drug-drug-interaction, PK, pharmacokinetic.

### 3.3 Follow-up analysis

A total of 13 clinical events were detected in this cohort (6.5%) that were possibly associated with the identified pDDIs. The majority were bleeding events (12 cases). One case had a prolonged QTc interval in the electrocardiography. Short narratives are listed in the supplement for each individual case.

Of note, 11 bleeding events were additionally detected in patients with AF, who had no underlying pDDI to modify the bleeding risk. Six patients had a thromboembolic event (myocardial infarction n = 1, pulmonary embolism n = 1, cerebral infarction n = 3, thrombotic, incomplete occlusion of the arteria femoralis communis). However, in those events we did not identify any association with pDDIs that could have increased the risk.

### 3.4 Comparison Lexicomp database vs. expert review analysis

Supplementary Table S1 presents the results of the Lexicomp database compared with each individual expert evaluation for the 20 randomly selected patients. There was considerable variability in the experts’ assessments of pDDIs. Furthermore, their results differed relevantly from the raw data obtained from the Lexicomp database.

## 4 Discussion

This study investigated the most common and the most severely graded pDDIs of patients with AF in the ED. To the best of our knowledge, this is the first study to investigate this specific group of patients. We identified 2085 pDDIs overall, of which 48 (2%) were classified with the highest severity (avoid combination), and 318 (15%) with severity D (consider therapy modification). About every fifth patient had at least one contraindicated drug combination, while 70% of patients had at least one drug combination for which a therapy modification is recommended. Hence, on average drug modifications may have to be considered in almost every AF patient. Interestingly, in the ED, patients received a median of 3 additional drugs (IQR 2–4), which led to a more than 3-fold increase in the number of pDDIs. This highlights the importance of considering interaction potential of newly prescribed drugs in this environment.

The number of different drugs a patient takes is the most relevant risk factor for pDDIs and ADRs (Wagh et al., 2019; Khan et al., 2019). Although there is no generally accepted definition of polypharmacy, most authors define it as a concomitant intake of 5 or more systemically active drugs (Masnoon et al., 2017/10; Jörgensen et al., 2001; Linjakumpu et al., 2002). In that context, the number of drugs correlated very well with the number of pDDIs (correlation coefficient 0.87 (p < 0.0001)). In our cohort, the median number of drugs was 5 (IQR 3–7) per patient before admission to the ED, which is comparable to a general ED patient population and means that most patients in this study fulfilled the criteria of polypharmacy even before the ED admission (Letinier et al., 2022). The comorbidities of the included patients were well in accordance with typical comorbidities of patients with AF, such as arterial hypertension, hyperlipidemia, and coronary artery disease (Hindricks et al., 2021; Gutierrez and Blanchard, 2016). Furthermore, the median age of patients with AF in our study (median 71, IQR 61–77) was similar to other large AF trials (Fromm et al., 2015; Lopes et al., 2018; Guimaraes et al., 2019; Bouida et al., 2019).

However, apart from this quantitative analysis, a qualitative analysis may provide more insight and offer important lessons for future patients with AF and treating healthcare professionals.

CDSSs facilitate a quick overview of pDDIs. Nevertheless, clinicians need to be aware of the disadvantages of these programs, as they may often be over-alerting, show interactions of low clinical relevance, or lack sensitivity and specificity (Kawamanto et al., 2018). Our expert review further emphasized the challenges of pDDI evaluation. Six independent experts assessed the most frequently occurring X- and D-graded pDDIs identified in this study population using resources of their own choice. However, the experts’ severity grading only considered two of these pDDIs (dronedarone – vernakalant; amiodarone – ibutilide) to be clinically relevant with a median severity grading of 3 (equals D or modify therapy). In all other cases, the experts found that the pDDIs were of less clinical importance and downgraded their severity. However, it is important to note that the variability of the experts’ gradings was remarkable. Furthermore, the experts’ analysis of 20 randomly selected patients revealed a large discrepancy in the number of identified pDDIs, both compared with the Lexicomp database that was used in this study and compared with the assessment of the other experts. The observed variability, even among experienced professionals, provides further evidence that the best possible pharmacological therapy for patients with polypharmacy is a major challenge. A multidisciplinary team approach that considers individual patient factors (e.g., medical history, laboratory parameters, frailty scores, pharmacogenetics, etc.) and a case-by-case evaluation may provide the best possible care for patients. Our data also show that the use of CDSSs alone may not be sufficient and may only support healthcare professionals in their decision-making process.

Importantly, several pDDIs that were identified in this study are subject to limitations. For instance, the combination of low-molecular weight heparin and DOACs may be the result of the applied methodology. We have analyzed drug combinations that occurred during the entire ED visit, which usually spans from a few hours to a maximum of 24 h. Thus, while patients may have received a low-molecular weight heparin first (e.g., after first diagnosis of AF), they may have been switched to a DOAC for long term therapy after an adequate time gap, or *vice versa*. However, in the Lexicomp database this combination would have been counted as an X-graded (avoid combination) pDDI according to the applied methodology. This specific methodological limitation in studies analyzing pDDIs is well-known (Bakker et al., 2022) and must be considered when interpreting the data. In this study, this very combination accounts for 9 of the X-graded pDDIs. Another example of such limitations is the combination of tiotropium and ipratropium, both muscarinic antagonists with anticholinergic effects, which are both applied by inhalation. Therefore, systemic side effects are unlikely to occur (Lipworth, 1996; Sadiq et al., 2021; Rau, 2005). However, most CDSSs do not differentiate between routes of drug administration. Furthermore, such drug combinations may be the result of different product availability in different hospitals, especially in the setting of emergency medicine. Again, the time gap between the intake of those two substances needs to be considered, which, however, was beyond the scope of this study.

In our follow-up analysis, we attempted to obtain a real-world estimate of the prevalence of ADRs, which might be associated with pDDIs. This retrospective analysis bears relevant limitations, because there was no systematic assessment of clinical consequences. Thus, one must exercise caution, when interpreting the numbers of the observed events. Expectedly, bleeding incidences occurred quite frequently with 12 cases (6%) in patients with respective pDDIs that increase the bleeding risk. There was one case of QTc prolongation in a patient, who took three substances that may prolong the QTc interval. However, this ECG was performed after catheter ablation, which is also known to cause a transient prolongation of the QTc (Nguyen et al., 2021). This example highlights the difficulty in ascribing clinical events to underlying risks, such as pDDIs.

Amiodarone is an established therapeutic option for AF (Hamilton et al., 2020), but it bears significant interaction potential: (i) inhibition of cardiac potassium channels (hERG-channel) for QTc prolongation, (ii) inhibition of various cytochrome P450 (CYP) enzymes and (iii) of P-glycoprotein (Hamilton et al., 2020; Harder and Thürmann, 1996; Taylor, 2002). In our study, amiodarone was involved in 113 X- and D-graded pDDIs and overall was the most frequently involved interacting drug. In 7 of the 13 patients with clinical events, amiodaron was one interaction partner. Another study which included elderly patients in the ED, reported comparable results: amiodarone was involved in numerous pDDIs and contributed to most contraindicated drug combinations (Letinier et al., 2022). Beside QTc prolongation, increased risk for bleeding in combination with anticoagulants was the most common pDDI (Table 4). In particular, the combination of amiodarone and anticoagulants poses an increased risk of major bleeding (Harder and Thürmann, 1996; Shurrab et al., 2023; Gronich et al., 2021; Daiichi Sankyo Europe GmbH, 2015; Boehringer Ingelheim, Pradaxa, 2009). Amiodarone increases the exposure to edoxaban by approximately 40% (Daiichi Sankyo Europe GmbH, 2015; Steffel et al., 2021), and to dabigatran by up to 60% via inhibition of permeability glycoprotein (P-gp) (Boehringer Ingelheim, Pradaxa, 2009; Steffel et al., 2021). In a recent study with 86,679 elderly patients (>66 years) combined use of amiodarone and DOACs increased the odds ratio for major bleeding to 1.53 (95% confidence 1.24–1.89) compared to intake of DOACs alone (Shurrab et al., 2023). Furthermore, a recent nationwide Belgian study in 193,072 patients with AF treated with DOACs investigated the concomitant use of P-gp/CYP3A4 inhibitors and inducers. Thereof, 46,194 (23.9%) received an inhibitor and 2,903 (1.5%) an inducer of either P-gp or CYP3A4. The concomitant use of P-gp/CYP3A4 inhibitors was associated with a higher bleeding risk (aHR 1.24, 95% CI, 1.18–1.30) and higher all-cause mortality (aHR 1.07, 95% CI, 1.02–1.11), while the use of P-gp/CYP3A4 inducers was associated with a higher risk to develop stroke (aHR 1.31, 95% CI, 1.03–1.68). (Grymonprez et al., 2023) These data should raise awareness of healthcare professionals.

The combination of amiodarone and propofol is frequently used if electrical cardioversion is necessary. Both substances exert a risk for QTc prolongation (Scalese et al., 2016). However, all patients subjected to undergo electrical cardioversion are monitored with regards to their electrocardiogram and vital signs. Therefore, possible arrhythmias and/or asymptomatic QTc prolongations would be diagnosed immediately. Although rare, Torsade de Pointes arrhythmia may be life-threatening and treating healthcare professionals should be aware of other risk factors, such as concomitant medication, hypokalemia, hypomagnesemia, long QTc at baseline, or a low heart rate (Abrich et al., 2017). Possible alternatives that do not prolong the QTc time include esketamine and/or midazolam (Woosley et al., 2024). There were no reports of prolonged QTc in electronic health records following the combined use of propofol and amiodarone in our cohort. However, within in this retrospective study, we did not systematically investigate QTc intervals. The only study describing prolongation of the QTc following propofol was conducted in critically ill patients, who likely received higher doses of propofol for a longer treatment duration. Hence, a direct comparison may not be possible. However, in the study by Scalese et al. the QTc interval increased by 30.4 ± 55.5 ms and 43.8% had a QTc>500 ms (Scalese et al., 2016).

Other drugs that bear a significant risk of QTc prolongation are antidepressants, specifically selective serotonin reuptake inhibitors (SSRIs) including escitalopram, fluoxetine, fluvoxamine, and sertraline (Funk and Bostwick, 2013). This specific class of drugs is of particular interest because 20%–40% of patients with AF suffer from depressive symptoms (Manolis et al., 2023). Furthermore, there is a possible association between depression and/or depressive symptoms and the risk of AF (RR 1.15, 95% CI, 1.03–1.27) and additionally the risk of AF increases with the use of antidepressants (RR 1.16, 95% CI, 1.07–1.25) (Fu et al., 2022). Interestingly, an Australian study that was conducted between 2014 and 2018 investigated the most common interacting partners with DOACs. The most frequent interactions partners were SSRIs/SNRIs (14.8%), followed by NSAIDs (9.7%), calcium channel blockers (8.8%) and amiodarone (6.5%). (Bezabhe et al., 2020) Of note, all drugs that reduce serotonin concentrations in platelets, like SSRIs and selective serotonin/noradrenalin reuptake inhibitors (SNRIs), may increase the risk of bleeding (Laporte et al., 2017). Bixby et al. discussed that SSRIs with a low (e.g., sertraline, fluoxetine) to intermediate (e.g., citalopram, venlafaxine) receptor affinity may not be associated with an increased risk of bleeding (Bixby et al., 2019). Furthermore, the use of non-serotonergic alternatives may reduce the bleeding risk (Bixby et al., 2019; McCloskey et al., 2008). However, the available data is somewhat inconsistent and further studies are still required to confirm these recommendations (Na et al., 2018; Carvalho et al., 2016). Nonetheless, the concomitant use of SSRIs with DOACs was associated with an increased risk of major bleeding (incident rate ratio 1.33; 95% CI, 1.24–1.42) in 42,190 patients with AF. The increased risk was maximal in the first 30 days (IRR 1.74; 95% CI, 1.37–2.2) and remained significantly elevated for up to 6 months (Rahman et al., 2024). In addition, a meta-analysis by Machado et al. in a population of 279,540 anticoagulated patients with concomitant use of SSRIs showed an increased risk of major bleeding (RR 1.33, 95% CI, 1.06–1.66) (Machado et al., 2023). In our cohort the combination of low-dose acetylsalicylic acid with an anticoagulant was frequent. However, given recent guidelines the prescription of anticoagulants replaces platelet inhibitors in most patients (Collet et al., 2021). Therefore, we believe that a critical appraisal of long-term medication is necessary in patients with AF, who will receive long-term anticoagulation. This is especially true with regards to amiodarone, antidepressants, NSAIDs and other platelet inhibitors.

Simvastatin, a CYP3A4 substrate was involved in numerous pDDIs. These pharmacokinetic interactions could easily be prevented by using statins that are not metabolized by CYP enzymes, or at least to a lesser extent, e.g., rosuvastatin or atorvastatin (Jones et al., 2003). A pharmacovigilance study of statins compared the risk of rhabdomyolysis. Among 10,657 reports between 1995 and 2022, 51.2% caused or prolonged hospitalization and 3.5% were fatal. Age ≥75 years (OR 1.82, 95% CI, 1.74–1.90) and male sex (OR 1.87, 95% CI, 1.80–1.95) were relevant risk factors. The risk of rhabdomyolysis was higher in patients with respective drug-drug interactions (OR 12.64, 95% CI, 11.64–13.7) and simvastatin had the highest risk of all statins (Montastruc, 2023).

Although pDDIs may be less relevant in the fast-paced environment of the ED, in stable patients, a brief medication analysis would be feasible and useful to avoid unnecessary potential harm and healthcare costs.

## 5 Limitations

This study has several limitations. Beside the above-mentioned methodological issues with the time-gap of drug intake and their respective duration of effects, the retrospective character of the study and the non-systematic evaluation of clinical endpoints in relation to the pDDIs must be mentioned. Patients from only one tertiary care hospital ED were included, which may limit the generalizability of the results. The analysis was limited to the ED stay and did not include changes to the medication during a possible hospital stay. Another concern is the specific pharmacological property of amiodarone with its very long half-life of up to 100 days after withdrawal of long-term therapy (Hamilton et al., 2020; Taylor, 2002; Latini et al., 1984). Consequently, amiodarone may affect drug metabolism even months after its last administration, which one would have to consider when performing pDDI analysis. A single database was used to identify pDDIs. Furthermore, therapy adherence of patients with regards to their long-term medication was not analyzed within this study. In the follow-up analysis the occurrence of minor bleedings may be underestimated because they are not always mentioned in the electronic notes. Furthermore, QTc was not systematically quantified. Moreover, the access to the electronic health record of the patients was limited to the hospitals of Vienna.

## 6 Conclusion

Healthcare professionals should be wary of pDDIs in patients with AF, especially with regards to additive effects on bleeding risk, QTc prolongation and risk for myopathy. Modification of long-term therapy should be considered to prevent pDDIs with AF-specific medication. This may influence the choice of long-term therapy with special regards to antidepressants or statins and should trigger deprescribing of unnecessary drugs.

## Data Availability

The original contributions presented in the study are included in the article/Supplementary Material, further inquiries can be directed to the corresponding author.
