# Supplementary material for: Drug-drug-interactions in patients with atrial fibrillation admitted to the emergency department
Source: Front Pharmacol. 2024 Oct 15;15:1432713. doi: 10.3389/fphar.2024.1432713 (PMC11538323; doi:10.3389/fphar.2024.1432713)
Supplement: Supplementary file 1 [file DataSheet1.docx]

**Supplementary materials**

Case 1: edoxaban – metamizole, amiodarone – edoxaban

A 59-year-old female patient with a history of hepatocellular carcinoma, discontinued edoxaban due to severe gum bleeding. Her co-medication included a regimen of metamizole (500mg tablets, 2-2-2), which may increase the bleeding risk of edoxaban. Furthermore, 1.2g of amiodarone were given at the ED visit, which may increase the serum concentrations of edoxaban. However, the bleeding incident was approximately three months after the ED visit and therefore an association seems unlikely.

Case 2: amiodarone - rivaroxaban

An 83-year-old male experienced a gastric bleeding event, while on rivaroxaban and amiodarone. He experienced such a bleeding incident previously without taking any anticoagulants.

Case 3: amiodarone - rivaroxaban

This 85-year-old male experienced a minor bleeding event (epistaxis) while taking rivaroxaban and amiodarone. His medical history is complicated by a diagnosis of multiple myeloma.

Case 4: acetylsalicylic acid - enoxaparin

A 79-year-old female had an active major bleed from the axillary artery, diagnosed in a CT scan. The bleeding was associated with a fall and subsequent fracture under concurrent treatment with enoxaparin and acetylsalicylic acid.

Case 5: acetylsalicylic acid - edoxaban

A 79-year-old female experienced epistaxis (minor bleeding) while being treated with edoxaban and acetylsalicylic acid.

Case 6: dabigatran - metamizole

A 65-year-old female experienced a minor rectal bleeding. She was treated with dabigatran and metamizole as needed, which increases the risk of bleeding.

Case 7: DOACs – amiodarone, phenprocoumon – amiodarone

An 81-year-old male with minor bleeding events on multiple anticoagulants (edoxaban, apixaban, rivaroxaban, phenprocoumon) during therapy with amiodarone.

Case 8: citalopram – edoxaban

A 75-year-old female experienced a major bleed from the gastroduodenal artery. At the time of the event, she was treated with citalopram and edoxaban.

Case 9: amiodarone – apixaban, apixaban – clopidogrel

An 84-year-old male underwent a tooth extraction followed by post-interventional bleeding. At the time of the intervention, the patient was taking clopidogrel, apixaban and amiodarone.

Case 10: amiodarone - phenprocoumon

A 79-year-old male had recurrent, minor, rectal bleeding episodes, both prior and during treatment with phenprocoumon, amiodarone, and later apixaban.

Case 11: amiodarone – sertraline, amiodarone – quetiapine, quetiapine – sertraline

A 50-year-old male presented with a prolonged QTc interval of 521ms post ablatio. His medication regimen included amiodarone, sertraline and quetiapine. However, QTc intervals may be prolonged in the post-ablatio-period without the influence of drugs.

Case 12: acetylsalicylic acid - sertraline

A 91-year-old male experienced a major gastrointestinal bleed while on acetylsalicylic acid and sertraline.

Case 13: escitalopram – rivaroxaban

A 70-year-old female presented with minor epistaxis while being treated with escitalopram and rivaroxaban.

Table S1: Potential drug-drug-interactions identified either by the Lexicomp database or by expert reviews for 20 randomly selected patients

|  | **Overall  (X-, D-, C-, B-, A-pDDIs)** | **X-pDDIs** | **D-pDDIs** | **C-pDDIs** | **B-pDDIs** | **A-pDDIs** |
| --- | --- | --- | --- | --- | --- | --- |
| **Lexicomp** | 210 | 4 | 23 | 168 | 15 | 0 |
| **Expert 1** | 180 | 15 | 0 | 162 | 0 | 3 |
| **Expert 2** | 53 | 3 | 7 | 32 | 4 | 7 |
| **Expert 3** | 71 | 8 | 4 | 52 | 3 | 4 |
| **Expert 4** | 25 | 0 | 1 | 14 | 0 | 10 |
| **Expert 5** | 34 | 2 | 19 | 4 | 0 | 9 |
| **Expert 6** | 216 | 3 | 6 | 71 | 134 | 2 |
| **median Expert rating (IQR)** | 62 (25-152.75) | 3 (2.25-6.75) | 5 (1.75-6.75) | 42 (18.5-66.25) | 1.50 (0-3.75) | 5.50 (3.25-8.5) |

Table S1 presents the number of potential drug-drug interactions for 20 randomly selected patients identified either by the Lexicomp database (raw data) or by individual experts. The results are sums for all X-, D-, C-, B-, and A-rated interactions of all 20 patients. The last row shows the median scores of the six experts including quartiles.

A = unknown interaction, B = no intervention required, C = monitor therapy, D = modify therapy, X = avoid combination

Figure S1: Correlation of drugs and pDDIs. pDDI = potential drug-drug-interaction
